# Scientific counterfactuals as make-believe

**DOI:** 10.1007/s11229-022-03949-8

**Published:** 2022-11-10

**Authors:** Noelia Iranzo-Ribera

**Affiliations:** 1 Department of Philosophy, University of Birmingham, ERI Building, Edgbaston, Birmingham, B15 2TT UK; 2 Department of Philosophy and Religious Studies, Utrecht University, Janskerkhof 13, Utrecht, 3512 BL The Netherlands

**Keywords:** Counterfactuals, Scientific reasoning, Fiction, Make-believe

## Abstract

Counterfactuals abound in science, especially when reasoning about and with models. This often requires entertaining counterfactual conditionals with nomologically or metaphysically impossible antecedents, namely, counternomics or counterpossibles. In this paper I defend the *make-believe view of scientific counterfactuals*, a naturalised fiction-based account of counterfactuals in science which provides a means to evaluate their meanings independently of the possibility of the states of affairs their antecedents describe, and under which they have non-trivial truth-values. Fiction is here understood as imagination (in contrast with its most typical association with falsity), characterised as a propositional attitude of pretense or ‘make-believe’ (Walton 1990). The application of this theory to scientific counterfactuals makes their evaluation a game of make-believe: a counterfactual is (fictionally) true iff its antecedent and the rules of the game prescribe the imagining of its consequent (Kimpton-Nye 2020). The result is a practice-based account of counterfactuals and counterfactual reasoning in science which incorporates insights from theoretical and experimental analytic philosophy as well as cognitive science. This way, the make-believe view of scientific counterfactuals shows that the evaluation of scientific counterfactuals is none other than a question of scientific representation in disguise.

## Introduction

My goal in this paper is to positively motivate a practice-based analysis of counterfactuals in science: what I call the *make-believe view of scientific counterfactuals*. This is a fiction-based account, which exploits the connection between counterfactuals and fiction already explored by Kim and Maslen (2006), Kimpton-Nye (2020), McLoone (2019), and Wilson (2021). However, it differs from these accounts in some respects: the motivation for fictionalism about counterfactuals, its scope, and the morals drawn.

Part of my motivation is naturalistic; I want to give an account of counterfactual reasoning that reflects how scientists use counterfactuals in practice. In Sect. 2 I identify two key features of such practice-based characterisation of counterfactuals and counterfactual reasoning in science: scientific counterfactual reasoning is none other than model-based reasoning (Sect. 2.1), and counterfactuals in science are non-vacuous (Sect. 2.2). This leads to two desiderata for a practice-based account of scientific counterfactuals: it ought to capture their connection to models, and it ought to capture their non-vacuity. I shall call the former **connection to models**, and the latter **non-vacuity**.

Furthermore, an account of counterfactuals in science which takes scientific practice at face value is one that results from a pluralist enterprise. Despite the solid institutional walls between disciplines, the philosophical study of the meaning of counterfactuals shouldn’t be disentangled from the cognitive study of counterfactual reasoning. Although this empirical turn in the philosophical analysis of counterfactuals is very recent, it is slowly gaining momentum. Woodward’s latest book *Causation with a Human Face*, for instance, revolves around the central idea that “*causation* and *causal cognition* need to be understood together” (2021, p. 1). There, he advocates a multidisciplinary approach to causation: “when it comes to causation, the armchair philosopher who appeals to intuitions, the experimental philosopher, and the empirical psychologist are in the same business, at least in many respects” (2021, p. 21). All three groups “should be understood as advancing empirical claims about patterns of causal judgement. The intuitions of the armchair philosopher have, in this respect, no special authority” (2021, p. 21). In the same spirit, while philosophers and cognitive psychologists pursue different programs regarding counterfactuals, there is a big area of overlap across these which a comprehensive study of counterfactuals—and especially of counterfactuals *in science*—shouldn’t overlook. This is why in Sect. 3 I review both the standard philosophical account of counterfactuals, namely the Lewis-Stalnaker similarity analysis (Sect. 3.1), as well as what I consider cognitive psychology’s best theory of counterfactual reasoning—the mental models view—developed mainly by Johnson-Laird (1983); Johnson-Laird and Byrne (1991), Byrne and Johnson-Laird (2009) (Sect. 3.2). I consider each with respect to the desiderata of **connection to models** and **non-vacuity**. Section 3.3 brings these analyses together and identifies a connecting theme between the two desiderata: the (scientific) imagination.

Section 4 contextualises, presents, and gives examples of the *make-believe view of scientific counterfactuals*, which is my fiction-based approach to counterfactuals and counterfactual reasoning in science. By linking counterfactuals’ antecedents to models, which are props in games of scientific make-believe, a clear connection can be established with **connection to models**. And by making counterfactual evaluation a matter of which fictional truths are propositions that are to be imagined, metaphysics doesn’t get in the way of semantics, so **non-vacuity** is respected. Section 5 concludes.

## Counterfactuals in science

A counterfactual is a subjunctive conditional whose antecedent is assumed to be false, either because it *did not* occur or because it *could not* occur. We might call the former ‘regular counterfactuals:’ those counterfactuals with antecedents which describe unactualised possibilities, that is, states of affairs which were once possible but did not take place. “If the coffee milk had been preheated it would not have curdled” is one such example. In the second group we find what philosophers typically call ‘counternomics’ and ‘counterpossibles.’ What these have in common is that they all have impossible antecedents, that is, their antecedents describe states of affairs which could not have occurred. The name ‘counternomics’—or ‘counterlegals’—is reserved for those counterfactuals with nomologically impossible antecedents, that is, antecedents which describe states of affairs which the actual laws of nature don’t permit. ‘Counterpossibles’ are counterfactuals with logically, mathematically, or metaphysically impossible antecedents. Hence, they can be respectively called ‘counterlogicals,’ ‘countermathematicals,’ or ‘countermetaphysicals.’

The focus of this paper is what I call ‘scientific counterfactuals,’ namely, counterfactuals that feature in scientific practice, that is, counterfactuals that are involved in explanations or predictions about the natural world. In the following sections, I identify their relevant features.

### Counterfactuals and scientific models

Scientific reasoning is full of counterfactuals. Below there is a sample list of counterfactual claims we oftentimes encounter:

- **SEA LEVEL:** ‘If concentrations of greenhouse gases had stabilised in the last decade, the North Sea level would have continued to rise.’
- **CORAL REEFS:** ‘If atmospheric $\text {CO}_{2}$ had remained at preindustrial levels, there would be less bleaching of the world’s coral reefs’ (List, 2022, p. 194)
- **IDEAL PENDULUM:** ‘If pendulum *X* (an actual pendulum) were a simple pendulum, then for small swings its period *T* would only depend on its length *l* and the gravitational acceleration *g*’ (adapted from Godfrey–Smith, 2009, p. 168)
- **H-BONDS:** ‘If water had not had intermolecular hydrogen bonding, then it would have been a gas at room temperature.’ (adapted from Tan, 2017, p. 959)
- **BOHR ATOM:** ‘If atoms were Bohr atoms, then an electron’s angular momentum *L* in the ground state would have been observed at $L=\hbar$’ (Tan, 2019, p. 48)
- **SPACE TIME:** ‘If space-time had had a dimension $\ge 4$, the planetary orbits would have been unstable’ (adapted from Woodward, 2003, p. 220; 2018, p. 123)

Note that SEA LEVEL and CORAL REEFS are regular counterfactuals, while the rest are all either counternomics or counterpossibles. But what do all of these counterfactuals have in common? First, counterfactuals in science appeal to scientific models. That is, their antecedents make reference to scientific models—they are a conjunction of all of a model’s assumptions—and their consequents are the predictions that follow from these. A minimal notion of ‘model’ is here adopted: models are small sets of propositions interpreted by some person to be about something. The ‘predictions’ captured by the consequents are those propositions entailed by the propositions that make up the model’s description together with relevant information that is extraneous to the model in question. A more detailed characterisation of this connection between models and counterfactual reasoning will be given in Sect. 4.2.1.

The idea that the content of a model can be captured via a counterfactual conditional, where the antecedent is a conjunction of all of the model’s assumptions and the consequent a conjunction of all the predictions that follow from these, is not new (see Godfrey-Smith, 2020; McLoone, 2021; Plutynski, 2006; Sober, 2011; Sugden, 2009). Examining which assumptions go into models brings up another typical feature of counterfactuals in science: many are counternomics or countermetaphysicals.^1^ This is not surprising considering the fact that models usually contain idealisations, abstractions, approximations, analogies, etc. Idealising away from problematic features is necessary in order to make phenomena mathematically and computationally tractable, and abstracting away from explanatory irrelevant features is needed for explanatory success, which often requires striking a balance between generality and depth (see Strevens, 2008). For instance, the simple pendulum model provides us with an equation which can be solved analytically under the assumption that the oscillations of the bob are small. The model incorporates other assumptions, all of which go against the laws of nature: the string is massless, the bob is a point mass, and there is no air resistance. IDEAL PENDULUM is thus a counternomic. So is BOHR ATOM: actual atoms are not described by Bohr’s model of the atom, which—contra observation—renders the hydrogen atom unstable. Some would say that it is metaphysically impossible for water to have been bonded differently, or for space-time to have had a different dimension than the actual; on this basis both H-BONDS and SPACE TIME might be deemed counterpossibles.^2^

### Counterfactuals and non-vacuous truth

Some of the most influential philosophical views on counterfactuals endorse vacuism about counterpossibles, the view that counterpossibles are vacuously true, that is, true in virtue of the fact that since their antecedents are metaphysically impossible they are *necessarily* false. Here one notably finds the semantic analyses by Stalnaker (1968) and Lewis (1973), as well as Williamson (2007)’s. For future reference let us call vacuism about counterpossibles the ‘vacuity thesis,’ following McLoone (2021). Despite its popularity within philosophy, the vacuity thesis is at odds with scientific practice. Even when antecedents describe impossibilities, scientists attribute the corresponding counterfactuals non-trivial truth values: they judge these to convey information on the basis of their truth or falsity. In addition to recent philosophical support (Jenny, 2018; McLoone, 2021; Tan, 2019), this ‘non-vacuity thesis’ has also received support from the first experimental study on counterpossible reasoning in science: Stuart et al. (2022) presented a group of self-described biologists with two counterpossible formulations, and asked them to judge whether these were true/false and why. Said judgements were found to be independent of the counterfactual antecedents’ (im)possibility.^3^ Since these results are important evidence for a naturalistic account of counterfactual reasoning, I will briefly recall the experiment they conducted. In said experiment, participants were presented with the logistic equation of population growth, and told that it would be used to model a population of real rabbits. The logistic equation is:

1 $$\begin{aligned} \frac{dN}{dt} = r N \Big (1 - \frac{N}{K}\Big ) \end{aligned}$$

Equation 1 is a differential equation that captures the evolution of population size *N* considering its intrinsic growth rate *r* and the habitat’s carrying capacity *K*. Rabbits are discrete, but Eq. 1 assumes them to be continuous. In view of this features of the logistic equation, the participants were asked to look at the following two counterpossibles (from McLoone, 2021, p. 12161), which for future reference I have labelled ‘SURVIVAL’ and ‘EXTINCTION’:

- **SURVIVAL:** If some population of rabbits satisfied the assumptions of the logistic equation, then the size of the population (*N*) would eventually be equal to the carrying capacity (*K*).
- **EXTINCTION:** If some population of rabbits satisfied the assumptions of the logistic equation, then the population would eventually go extinct.

Participants were first briefed about the differences between nomological and metaphysical impossibility, and asked whether the assumption that rabbits come in non-integer values was metaphysically impossible. After that, they had to assign truth-values to these two counterfactuals and justify their answers. The results show that a significant majority of participants judged SURVIVAL to be true and EXTINCTION to be false, and relied on the mathematical relation between the antecedent and the consequent to do so regardless of what they thought about the (im)possibility of their shared antecedent. It is precisely the fact that the (impossible) modal status of the states of affairs described by these counterfactuals’ antecedents is left out from counterfactual evaluation what ensures that all the obtained truths are non-vacuous. Clearly more experimental studies on the non-vacuity of counterfactuals with impossible antecedents are needed, but even this first study puts extra pressure on the vacuity thesis.

In short, in this section I have explored the scientific counterfactual space and introduced some assumptions regarding its characteristics: counterfactuals in science are connected to models and they are non-vacuous, especially those with impossible antecedents, which comprise a big subset thereof.

## The toolbox for counterfactual analysis

### A philosopher’s take: the similarity analysis

The meaning of counterfactual conditionals is often described using the similarity analyses developed almost simultaneously by Stalnaker (1968) and Lewis (1973).^4^ Their accounts construe counterfactual conditionals as factual, categorical statements, which are analysed via possible-worlds semantics, a framework which models different modal notions (necessity, possibility, and counterfactuality) on the basis of truth at possible worlds, where possible worlds are usually understood as a convenient way of speaking about how the actual world could have been.^5^

‘If I had struck this match, it would have lit.’ Intuitively, this counterfactual is true. However, had I struck this match in a room without oxygen, on a wet surface, or not energetically enough—so that there was not enough friction between the match head and the surface—this would not be so. Stalnaker and Lewis agreed that the context is here important: in order to assess the meaning of a counterfactual A  C we should look at just the relevant possible world (Stalnaker) or set of possible worlds (Lewis), which are those which are *closest* or *most similar* to the world at which the counterfactual under scrutiny is evaluated. Formally, A  C is true iff the counterfactual’s consequent (C) is true at every closest possible world at which the antecedent (A) is true.

What the notion of similarity precisely amounts to has been the focus of debates about the suitability of the similarity analysis for counterfactual evaluation. In the following I focus on Lewis’ account, for he proposed a system to weigh similarities between worlds against their differences in order to arrive at a notion of overall comparative similarity between those worlds (see Lewis, 1979, p. 472). As a matter of example, the first of these weights tells us to avoid big, widespread, diverse violations of law. The second recommends that one maximises the spatiotemporal region of perfect match of particular fact. And so on. Lewis’ similarity analysis has, with good reason, pervaded the philosophical psyche: even if sketchy, it provides a recipe to evaluate counterfactual possibilities in terms of *distance* to the actual world. However, it falls short of resources for a naturalised view of scientific counterfactuals like the one I am after. Here’s why.

Regarding **connection to models**, models are *not* like possible worlds. Possible worlds are complete, which means that in possible-worlds-based semantics all propositions are determinate with respect to truth. However, models are not complete: intuitively, models only enable the assignment of determinate truth-values to the relevant propositions, namely, those that fall within the scope of the model. Those that fall outside of a model’s scope are indeterminate, for the model does not include the facts that would make these propositions true or false (Currie, 1990; Salis & Frigg, 2020; Walton, 1990).^6^ Hence, possible-worlds-based semantics for counterfactuals do not naturally apply to scientific models.

In relation to **non-vacuity**, the Lewis-Stalnaker analysis of counterfactuals leads to the vacuity thesis: if a counterfactual’s antecedent (A) is *metaphysically* impossible, there is no possible world which satisfies A—there is no A-world—and so no closest A-world. The counterfactual is then trivially true. Why is the vacuity thesis problematic? First of all, trivially true counterfactuals are uninformative. In the Lewis-Stalnaker semantics counterpossibles turn out true in virtue of the fact that their antecedents are impossible, and so any consequent is logically implied. Therefore, the consequents of counterpossibles add nothing to their truth-value or meaning (Brogaard & Salerno, 2013, p. 642). For instance, ‘If wizards were green, they would photosynthesise’ is true because there are no wizards, and not because “it describes a substantive fact about what the wizards are like or which properties they have” (Tan, 2019, p. 34).^7^ Second, and more importantly, the vacuity thesis contravenes scientific practice, which as seen in Sect. 2 renders some counterpossibles non-trivially true and others false.^8^

One way to handle the vacuity of counterpossibles would be to reject those metaphysical views under which the vacuity thesis obtains as implausible or fringe. However, this would be an ad hoc move. Furthermore, the low prevalence of the vacuity thesis is challengeable, for the vacuity thesis obtains under a spectrum of modal views. These include, on the one hand, philosophers who think that these counterfactuals’ antecedents violate the essential nature of non-fundamental kinds. Tan (2019, p. 46), for instance, argues that it is a matter of metaphysical necessity that water is $\text {H}_{2}\text {O}$ and so it is composed of discrete molecules. As a result, any scenario where water’s microstructure is different than its actual one is metaphysically impossible. On the other hand, the vacuity thesis concerns those philosophers for whom the counterfactuals in Sect. 2 come out as metaphysically impossible due to the necessity of the laws of nature. Here we find dispositional essentialists, for whom the laws are metaphysically necessary contingent on the instantiation of the relevant natural properties or kinds, as well as modal necessitarians, for whom the laws of the actual world are the laws of all the possible worlds. Due to the fact that realising the antecedents of the counterfactuals in Sect. 2 would involve the violation of one or more laws of nature, these are counternomics. Given that the laws of nature are (conditionally or unconditionally) metaphysically necessary, these turn out to be counterpossibles as well.^9^

Recently, other responses to the vacuity thesis have been offered, which either extend Stalnaker–Lewis semantics with impossible worlds (see McLoone, 2021), or repurpose possible worlds using modal counterpart theory (see Hicks, forthcoming). While these meet the demands of **non-vacuity**, McLoone (2021, pp. 18–20) himself acknowledges his strategy is not precisely parsimonious, which is something which could also be said of Hicks’ account. Whether and, if so, how they meet **connection to models** would have to be analysed. I believe a third overlooked strategy, which meets both desiderata in a more straightforward way, is up for grabs.

### A cognitive psychologist’s take: the mental models theory

In cognitive science, the term ‘possibility’ is often used in a broader sense than it is used in philosophy. For instance, for Nichols and Stich (2003, p. 25) ‘possibility’ designates any description which doesn’t include any obvious logical contradiction. What they call the ‘Possible World Box’ (PWB) is a cognitive workspace which basically represents what the world would be like given some assumptions whose truth-value is unknown to us. The PWB is one of the cognitive mechanisms that underlie the capacity for pretence, which has been argued to be implicated in counterfactual reasoning (Goldman, 1992).

Counterfactuals not only have been and continue to be a controversial topic for philosophers, but also for cognitive scientists. Research in cognitive psychology has given rise to different theories about the cognitive process(es) that underlie the production and evaluation of counterfactuals. Alongside the view that counterfactual reasoning is a matter of calculating conditional probabilities, one also finds the view that human reasoning proceeds by means of simulating ‘mental models.’ Nowadays we call ‘mental models’ accounts those based on this idea (see e.g. Byrne & Johnson-Laird, 2009; Johnson-Laird, 1983; Johnson-Laird & Byrne, 1991). In Sect. 3.3 argue that they offer valuable insights with respect to counterfactual reasoning in science.

According to the proponents of mental models accounts, people understand conditionals by mentally simulating (im)possibilities (Kahneman et al., 1982). Counterfactual reasoning is thus none other than thinking about possibilities in the loose sense of the aforementioned PWB (see Byrne, 2016, p. 342), represented as small mental models of the situation. In everyday counterfactual reasoning people usually represent dual possibilities, reality and an alternative to it (see Byrne, 2005, 2007). This idea that when evaluating counterfactuals what people do is imagine situations in which these counterfactuals’ antecedents hold and then run mental simulations forward to see what happens in these imagined situations—leaving much else unchanged—is broadly in line with Lewis’ account. In fact, in *The Rational Imagination* Byrne (2005) shows that people tend to imagine worlds with the same natural laws, a result which is captured by Lewis’ first criterion of similarity of worlds (see Sect. 3.1).^10^ The idea that mental models are *imagined* counterfactual situations fits nicely with **connection to models**: in science, models are often understood as imagined systems—imagined economies, populations, gases, etc.^11^

What about **non-vacuity**? In mental models theory, whether a counterfactual is true is a matter of whether a certain consequent follows in our mental simulations of the situation in question. For more details on the theory’s account of the meanings of conditionals see Espino et al. (2020, pp. 1265–1266). What is of main interest here is the fact that this theory does not rely on the notion of conditional probability, and so the content of counterfactuals, some of which may have impossible antecedents, can be represented just as well as the content of indicative conditionals.^12^ In fact, in recent work Byrne (2022) concludes that mental models can be straightforwardly extended to counterpossibles: “[p]eople simulate impossible conjectures as if they were possible, relying on knowledge of reality to constrain their interpretation of them as true or false” (p. 19). This is because modal judgements about antecedents, i.e. judgements about how probable or similar to actuality a possibility is, do not preclude their mental simulation and evaluation of the resulting counterfactuals.^13^ This result is in line with the research outputs of the experiments undertaken by Stuart et al. (2022), who as seen in Sect. 2 have given some evidence that reasoners’ judgements of the truth-values of counterfactuals do not bear on their judgements about the (im)possibility of the counterfactuals’ antecedents.

In short, cognitive research in the mental models theory of counterfactual reasoning highlights the central role of the imagination, which is often acknowledged by philosophers of science working on modelling. In the mental simulation of the scenarios described in counterfactuals’ antecedents, their modal status plays no role, so the vacuity thesis can be sidestepped.

### Assembling the pieces: scientific models and the imagination

In Sect. 1 I identified two desiderata for a naturalised view of counterfactuals and counterfactual reasoning in science. The first, **connection to models**, tells us that it ought to respect the key role counterfactuals play in scientific modelling. The second, **non-vacuity**, is self-explanatory: most counterfactuals are either non-trivially true, or false.

With respect to **connection to models** both armchair and experimental philosophers of science agree on the connection between counterfactual reasoning in science and models, which have a pivotal and widespread use in science. In Sect. 3.1 I argued that models are *not* like possible worlds, for models are incomplete. How should we then conceptualise them? Looking at cognitive science and actual scientific practice, models look more like *imagined* scenarios which the antecedents of counterfactuals like those in Sect. 2 invite us to consider. Philosophers working on modelling in science have long emphasised the involvement of the imagination in modelling; models have been described as e.g. imagined objects (Frigg, 2010) or imagined images of the world (Morgan, 2004). Byrne (2005) has argued, contra folk belief, that the imagination is a *rational* cognitive faculty which allows reasoners to generally correctly evaluate the connections between counterfactuals’ antecedents and their consequents.^14^ This idea that “many people are rational, in the sense that they make normatively appropriate causal inferences and judgements much of the time” has also been discussed by Woodward (2021, p. 5). Having identified the imagination as key in counterfactual reasoning, we now need to fine-tune what type of imaginative process counterfactual reasoning in science is. Byrne (2005) leaves the characterisation of imagination quite open for day-to-day counterfactual reasoning: perception, or something like our comprehension of discourse, might be involved in mental modelling. Given that our focus here is on counterfactuals, which are expressed as propositions, I believe a *propositional* notion of imagination is the obvious candidate: we want to be able to speak of the content of counterfactuals as true or false. This choice is reinforced by the fact that counterfactuals in science, which often trade in impossible hypothetical scenarios, may be difficult to visualise. In this case, only a propositional type of imagination is able to render these intelligible.

Regarding **non-vacuity**, in Sect. 2 we have seen that in recent years some theorising against the philosophical orthodoxy of the vacuity thesis has been put forth by scientifically-minded philosophers. The idea that all counterfactuals with impossible antecedents may have non-trivial truth-values has now received empirical support from the research conducted by Stuart et al. (2022). It is precisely because of the non-triviality of their antecedents that informative consequents can be inferred from these. This non-vacuity results from abandoning the unfruitful idea that what makes a certain counterfactual true are some objective relations between matters of fact at some possible world which stands in some similarity relation to the actual world.^15^ Rather, what makes a counterfactual true is a matter of whether a counterfactual’s consequent (C) follows from its antecedent (A). This basic mechanism, which underlies the mental models account of conditionals (Sect. 3.2), can equally accommodate all types of counterfactuals: regular counterfactuals, counternomics, and countermetaphysicals. The task at hand is to make precise what ‘follow’ means here; I will do so in Sect. 4.2.1.

To sum up, a good account of counterfactuals and counterfactual cognition in science ought to pay attention to the connection with modelling practice, which emphasises the key role of the imagination—understood propositionally—in counterfactual evaluation. Furthermore, it ought to accommodate the empirical results which show that counterfactuals are evaluated on the basis of whether their consequents follow from their antecedents, an evaluation which is silent to the modal status of the states of affairs described by their antecedents. Luckily, there is one such account available, which I call ‘the make-believe view of scientific counterfactuals.’ This is a fiction-based account of counterfactuals, which closes the gap between traditional philosophical theories of counterfactuals and scientific practice, and reclaims counterfactuals as a rightful object of study for philosophers of science.

## Fictionalism about scientific counterfactuals

### Contextualising fiction

On the one hand, counterfactuals feature prominently in scientific models. On the other, counterfactual reasoning is an imaginative activity. When put together, these two observations suggest a view of models as “works of fiction” (Cartwright, 1983, p. 153). The idea that the notion of *fiction* might be relevant to the practice of model-building and scientific representation with models is not new. It was explored in a series of essays edited by Suárez (2009), as well as by those who advocate any version of what is nowadays known as *the fiction view of models*. In the following extend this view of scientific models to counterfactuals in science, and thereby propose a fiction view of counterfactual reasoning in science, the make-believe view of scientific counterfactuals. While I am not the first to propose a fictionalist view of counterfactuals (see e.g. Kim & Maslen, 2006; Kimpton-Nye, 2020; McLoone 2019; and Wilson, 2021), my account offers new insights with respect to the motivation, scope, and implications of fictionalism about counterfactuals in science. First, I introduce both the notion of fiction it uses (Sect. 4.1.1), as well as the fiction view of models it draws on (Sect. 4.1.2). Then, I develop the make-believe view of scientific counterfactuals and discuss in which respects it improves on the aforementioned literature (Sect. 4.2).

#### Fiction as imagination: make-believe

Prima facie, the word ‘fiction’ usually connotes *falsity*. The word has been ordinarily used to refer to a description which involves falsity or inaccuracy, and which is known to involve it by those who employ it. If the fiction is a proposition, it is understood as a deviation from truth, if it is an object, it signals a deviation from existence. The fact that fictions in this sense are introduced ‘deliberately’ or ‘purposefully’ is what distinguishes them from mistakes.^16^ This view of fiction as counterfeit, forgery, or fake has received many names, such as the *truth-conditional* sense of fiction (Suárez, 2009), or fiction as *infidelity* (Frigg & Nguyen, 2020, pp. 105–113).

However, this is not the only meaning of the word ‘fiction.’ Fiction also connotes *imaginings*, regardless of the accuracy of their content. This second sense of fiction, called *functional* (Suárez, 2009), or fiction as *imagination*, is the one I henceforth use. In particular, I borrow Kendall Walton’s (1990) understanding of fiction in his ‘pretense theory,’ the systematic study of what he calls ‘games of make-believe.’

Make-believe is “the use of (external) props in imaginative activities” (Walton, 1990, p. 67). The notion of prop must be understood loosely; anything which prompts one to imagine, ranging from stumps to paintings or texts, might qualify as a prop. Now, what sorts of imaginings are mandated by props is constrained by ‘principles of generation,’ the rules of what is to be imagined. These are largely ambiguous, and range from tacit knowledge and belief to logical rules of inference or mathematical knowledge. “[W]hat principles of generation there are depends on which ones people accept in various contexts” (Walton, 1990, p. 38), so make-believe is essentially a social activity.

As previously noted, props prescribe an attitude of “imaginative engagement” (Frigg, 2010, p. 110): when we say ‘It is fictional that *p*’ what we mean is that ‘It is to be imagined that *p*.’ What makes it fictional is “the attitude that the reader is expected to adopt towards it” (2010, p. 110). I follow Currie (1990, p. 20), McLoone (2019), and Salis and Frigg (2020) in characterising this attitude of imaginative engagement as *propositional*.^17^ In the game of make-believe triggered by Kafka’s *The Metamorphosis*, for instance, the reader is mandated to imagine *p* = *that Gregor Samsa finds himself transformed into a gigantic insect.* Propositions like *p* have the property of being ‘fictional.’ If a proposition is fictional with respect to a certain game of make-believe, we say it is a ‘fictional truth’ in that game of make-believe. Hence, *p* is fictionally true (f-true) in the game of make-believe prescribed by Kafka’s *The Metamorphosis*. Truths directly generated by the prop are called ‘primary,’ those indirectly generated by the prop and some rules of inference are called ‘implied’ (Walton, 1990, pp. 140–144). Importantly, imagining *p* does not commit one to its truth, even though make-believe is not incompatible with it. Therefore, truth-in-fiction is *not* a species of truth, even though fictional truths might ground truths.^18^

#### The fiction view of models

Scientists often reason about imaginary biological populations, neural networks, economies, or pendula, which are ordinarily used as stand-ins for the chunks of the world we investigate. The ‘fiction view of models’ is an umbrella term for those views of models which compare these imaginary objects to those of literary fictions, an analogy originally formulated by Godfrey-Smith in his paper ‘The strategy of model-based science’ (2006). I find this analogy helpful, for the reasons I now expose.

The main contemporary versions of the fiction view of models recognise the central role of the imagination in modelling practices, and build on Walton’s notion of make-believe as a social imaginative activity (Salis, 2020, p. 2). Its mechanics are the following: model descriptions are seen as props, which prescribe imaginings about either ‘model systems’ or directly about physical systems (which in the modelling literature are called ‘target systems’). The former view is called the ‘indirect fiction view of models’ because models represent their target systems indirectly, via the mediation of model systems (see e.g. Godfrey-Smith, 2009, or Frigg, 2010; Frigg & Nguyen 2020).^19^ A supporter of this view will e.g. defend that the ideal gas model prescribes imaginings about a model system, the ideal gas, which is taken to represent a target system, a real gas under normal conditions of pressure and temperature. The latter view is analogously called the ‘direct fiction view of models’ because the model description directly prescribes imaginings about a target system without the mediation of a model system (see e.g. Toon, 2012 or Levy, 2015). Back to the gas example, a supporter of the direct view will defend that the model description of the ideal gas prescribes imaginings of a real gas described somewhat differently from the way it really is.

Both fiction accounts of models face difficulties. The main hindrance of the indirect fiction view is the problem of transfictional propositions, namely, the problem of cashing out model-target comparisons on deflated views of models (see e.g. Godfrey-Smith, 2009, 2020 or Levy, 2015 for expositions of the objection). In fact, the direct fiction view was proposed as a reaction to the problem of transfictional statements: by eliminating the intermediate object which mediates the representation of the target system said problem does not arise. However, the direct fiction view of models faces a challenge of a different sort: it cannot accommodate targetless models—such as multi-sex populations (Weisberg, 2013, Chap. 7), the $\phi ^4$ in quantum field theory (Frigg, 2010), or Schelling’s famous segregation model (Schelling, 1971)—nor what Weisberg calls ‘generalised’ models (models of general phenomena, such as parasitism) and ‘hypothetical’ models (models of nonexistent targets, such as xDNA). In Godfrey-Smith’s words, direct fiction views “have to massage the phenomena quite a lot” (2009, p. 159). Toon’s (2012) response to these cases is to treat them as exceptions: model descriptions prescribe imaginings of target systems unless the models have no target, in which case they prescribe imaginings about fictional systems. However, this latter option inherits the problem of transfictional statements for targetless models. Levy’s (2015) response to the objection is to dismiss these cases as either only apparently targetless or as bits of mathematics. This poses an immediate question: under the assumption that the target will eventually be clarified, what is the model *about* until then? Consider the following counterfactual: ‘If neutralinos existed, they would only interact with the intermediate vector bosons.’ We don’t know whether these hypothetical particles of supersymmetry models will eventually be found, but we nonetheless treat models of these as entities in their own right. The main difficulty of the direct fiction view is the fact that it makes modelling parasitic on what is already known to exist. By making the definition of ‘model’ independent from modelers’ epistemic states, the indirect fiction view comes out as a more inclusive alternative.^20^

The aforementioned inclusivity results from the workings of the indirect fiction view: the indirect view cashes out modelling as a two-step process, where in the first step one investigates the model, and in the second one investigates whether the model bears on the world (and, if so, how this representational relation functions). This explains why the two notions of fiction—fiction as falsity and fiction as imagination—need not be mutually exclusive: fictions might still represent, beyond themselves, but crucially, it is in virtue of their imaginative nature and not this representational power that they are fictions. Translated to truth, this is the idea that when modelling one first *pretends* the truth of the claims implied by the model, and only afterwards considers whether these fictional truths map onto truths via one’s preferred theory of scientific representation, such as Frigg’s and Nguyen’s DEKI account of scientific representation (2016; 2020). Asking about the representational power of a model is then a question that is bracketed when investigating a model. I believe the characterisation of modelling as a two-step activity offers a good and unifying reconstruction of counterfactual reasoning in science.^21^ When reasoning with counterpossibles, one often first asks whether a consequent follows from the antecedent in question before examining how the content of the imaginary exercise in question relates to the world. Given that there don’t seem to be any relevant cognitive differences between regular counterfactual reasoning and counterpossible reasoning (see Sect. 3.2), it is only natural to characterise regular counterfactuals in the same way. This independence from knowledge of actual states of affairs afforded by the indirect fiction view of models thus enables a uniform treatment of counterfactuals, which the direct fiction view can’t offer. This is the main reason why I favour an indirect fiction view of models.

One key feature of the fiction view of models is that it brings imagination to the foreground. The ‘imaginary’ is a category which signals merely possible objects which have a constructed nature, so it captures the fact that even if models often describe mind-independent physical patterns, models are themselves *created* by scientists. Hence, and unlike abstract mathematical objects, models are candidates for concreteness, namely, “candidates for entering into causal relations with real objects.” (Godfrey-Smith, 2020, p. 159). Furthermore, the type of imagination involved in make-believe is not “an unbridled flow of free thoughts” (Frigg & Nguyen, 2020, p. 120). One worry here is that the central role of the imagination in modelling can be stressed without reference to fiction.^22^ Weisberg (2013), for instance, has an account that recognises the role of the imagination in modelling, yet at a high price: imaginary systems are treated as integral to the practice of modelling, but are not to be identified with the models themselves. Imaginary systems are thus part of the folk ontology of modelling, and this is so because he conceives of the imagination as primarily *imagistic*. But how can folk ontology be essential? I believe that this can be easily remedied by turning to a characterisation of the key imaginative engagement with models as essentially *propositional* instead of imagistic—but this is none other than what the fiction view of models proposes.

In the fiction view of models, model descriptions prescribe games of make-believe regimented by props and principles of generation, which in science are mathematical knowledge, laws of nature, and logical rules of inference. In short, reality-oriented principles and inferential tools. In make-believe there is no general recipe of what constitutes a principle of generation beyond the expectation that something like Walton (1990)’s ‘reality principle’ (pp. 144–150)—a principle which used in the context of modelling would instruct us to import actual theoretical information unless otherwise specified by the model’s description—or the ‘mutual belief principle’ (pp. 150–161)—under which the implied truths generated are the result of beliefs held by the scientific community—obtains.^23^ While principles of generation might arguably be perceived as vague, and so epistemically idle, this is only so because of their highly contextual form.

In the context of scientific inquiry, it is not only the content of games of make-believe that is constrained—or rather, guided—but also their scope: make-believe within the fiction view of models excludes modelling for merely recreational purposes (Godfrey-Smith, 2020, p. 160); scientific make-believe has epistemic aims such as explanation, justification, the drive of empirical discovery, or the enhancement of understanding.

### The make-believe view of scientific counterfactuals

#### Exposition

In the following, I expose my fiction-based view of counterfactuals in science, which draws on the fiction view of models in important respects. Before that, it is nonetheless paramount to dispel one potential concern straight away.^24^ In ‘The Scientific Imagination’ Salis and Frigg (2020) present counterfactual reasoning as a type of propositional imagination distinct from make-believe. According to them, the difference lies in the fact that even though counterfactual reasoning and make-believe share a minimal core, they differ in the additional elements which characterise them. These differences are, however, less clear-cut than they are presented as. The two distinctive elements of counterfactual reasoning, they argue, are *selectivity* and *reality orientation* (Salis & Frigg, 2020, pp. 32–34). Since these elements capture aspects of the Lewisian similarity relation (see Sect. 3.1), I believe they are none other than Walton’s principles of generation for make-believe, i.e. the reality principle and the mutual belief principle.^25^ At the same time, the distinctive feature of make-believe is that it is a *social activity* with a *normative aspect* (Salis & Frigg, 2020, pp. 35–36). Counterfactual reasoning in science is certainly social, for scientific knowledge is socially embedded: scientific communities set the limits of which games of make-believe are permitted. The normative aspect of make-believe goes via its connection to imagined models, as it will hopefully become clear shortly. The additional elements thus turn out to be less distinct than intended. As a result, counterfactual reasoning and make-believe are not mutually exclusive categories.

Having shown how make-believe makes room for counterfactual reasoning, let me now introduce the make-believe view of scientific counterfactuals. This view characterises counterfactuals in science via make-believe in the way suggested by Kimpton-Nye in his paper ‘Necessary Laws and the Problem of Counterlegals’ (2020): a counterfactual’s antecedent (A) serves as a prop in a game of make-believe. If the counterfactual’s consequent (C) is prescribed for imagination given A and the pertinent principles of generation, then the counterfactual in question is true within the fiction, or f-true (pp. 529–530). These are the semantics of the make-believe view of scientific counterfactuals. My view will build upon Kimpton-Nye’s account by fine-tuning its details so that the account fulfills the desiderata of **connection to models** and **non-vacuity**, which I identified as key to a naturalised account of counterfactuals in science.

Let us first look at **connection to models**. Throughout this paper I have argued that there is a tight connection between counterfactuals and models in science; consequently, the make-believe view of scientific counterfactuals builds upon the fiction view of models. Scientific counterfactuals are connected to scientific models via their antecedents, which appeal to more or less full-blown scientific models. Recall the counterfactuals IDEAL PENDULUM and BOHR ATOM from Sect. 2: the antecedent of IDEAL PENDULUM, ‘If pendulum *X* (an actual pendulum) were a simple pendulum...,’ alludes to the simple pendulum model. Similarly, BOHR ATOM’s antecedent, ‘If atoms were Bohr atoms...,’ makes explicit reference to Bohr’s model of the hydrogen atom. Other counterfactuals will have antecedents which will not be connected to well-established full-blown scientific models, but under the minimal characterisation of ‘model’ here adopted, where models are sets of fictional propositions that are interpreted by someone to be about something, their widespread acceptance and use is not required. This needs to be so in order to accommodate impossible counterfactuals, which encode states of affairs whose modelling might be in a more embryonic state. This is the case for counterfactuals such as H-BONDS and SPACE TIME, which respectively describe an alternative bonding structure for water and a different dimensionality for space-time.

One might worry that **connection to models** is unnecessarily strong; while granting the close relationship between scientific counterfactuals and models, a potential objection to the present account is that there might be cases where counterfactual reasoning in science is not model-based.^26^ Indeed, in some of the available accounts of scientific modelling the connection between theoretical activities and modelling in science is looser than the one I suggest. Weisberg (2007), for instance, distinguishes modelling, which he characterises as *indirect* representation, from what he calls ‘abstract direct representation’ (ADR). In his account, the periodic table is *not* an instance of modelling because chemical properties of chemical elements such as valency or atomic weight are *directly* represented. But what does it mean to directly represent a target system? Weisberg differentiates theoretical activities on the basis of the *actions and intentions of theorists* (2007, p. 222) rather than the *products of theorising* (2007, p. 228). The key distinction aims to be whether it is the primordial aim of the theorist to “analyze and represent the properties of a real-world phenomenon, suitably abstracted, in the first instance” (ibid., p. 222). If so, we have an instance of ADR, otherwise said representational object is a model. On Weisberg’s account, all of the counterfactuals introduced in Sect. 2.1, except for perhaps H-BONDS and SPACE TIME, describe instances of ADR rather than modelling. But these, such as BOHR ATOM, certainly appeal to models, scientists would complain. My main reason for rejecting Weisberg’s taxonomy of theoretical activities is that the make-believe view of scientific counterfactuals aims to be an account which takes scientific practice at face-value, and it is precisely the products of theorising, namely, those non-imagistic representations of real-world systems containing idealisations, abstractions, and/or approximations, which practitioners usually call models. My proposed characterisation of models accounts for this feature of practice, and as explained in Sect. 4.1.2 I consider this inclusivity a blessing rather than a curse.^27^

Having hopefully dispelled the concern that **connection to models** is unnecessarily strong, let us now assess how the present view fares with respect to **non-vacuity**. The output of non-vacuous truths results from the conceiving of counterfactual evaluation as a matter of whether a counterfactual’s consequent follows from its antecedent and a set of principles of generation. The principles of generation involved in scientific counterfactual reasoning are those involved in make-believing with scientific models (see Sect. 4.1.2), namely, laws and other physical principles, mathematical knowledge, and rules of logical inference. What is distinctive about counterfactual reasoning in science versus day-to-day counterfactual reasoning is that the former presupposes the figure of a competent reasoner, namely, a member of the relevant scientific community. This competent user finds herself in a particularly good epistemic position, or at least the most optimal one, to identify the relevant principles of generation which a certain instance of counterfactual reasoning requires, and thus minimises the risk of faulty inferences.

The make-believe view of scientific counterfactuals also inherits the constraints on the scope of application of the fiction view of models. That is, make-believing in scientific contexts mostly has epistemic roles; other purposes such as recreation, art, or planning are excluded. Whether, and if so how much, the imagination contributes to such epistemic outcomes is a focus of debate in the literature on the (scientific) imagination.^28^ Notwithstanding the lack of consensus regarding the precise contribution of the imagination on the epistemic character of its outputs, we can nonetheless discuss the roles these play. The examples provided in Sect. 2.1 can help identify some epistemic uses of counterfactuals in science: to convey explanations (a function of all the counterfactuals there listed), to drive empirical discovery (as is the case for H-BONDS), to justify the reliance on our current scientific theories (as is the case for BOHR ATOM), or to enhance tractability (such as in the case of idealisations like IDEAL PENDULUM). This list is not exhaustive, but only illustrative.

Regarding **non-vacuity**, reliance on make-believe has the further advantage that counterfactual evaluation is not defied by impossibilities. The make-believe view of scientific counterfactuals puts modal judgements about the content of propositions on hold: one need not believe or know that a certain counterfactual is true in order to engage in a game of make-believe triggered by its antecedent and the pertinent rules of generation, so the purported impossibility of a counterfactual antecedent makes no difference for the purposes of counterfactual reasoning and evaluation. This is in line with the theorisation by McLoone (2019, 2021), the results of experimental philosophical experiments presented by Stuart et al. (2022), and the results of cognitive psychological experiments (see Sect. 3.2).

One very reasonable worry about fiction-based accounts of counterfactuals is that while truth-in-fiction might suffice to evaluate a counterfactual’s fictional meaning, it cannot help evaluate counterfactual claims extraneous to the fiction. To some extent, this is true: what happens in the fiction stays in the fiction. However, the make-believe view of scientific counterfactuals allows one to export what one has learnt about the fictional system outside of the game of make-believe via a suitable theory of scientific representation for models. It is when representation of real-world systems by imagined systems is successful that we can say that fictional truths about imagined systems ground truths about real-world systems. This is where **connection to models** and **non-vacuity** converge: the make-believe view of scientific counterfactuals shows that the question of the semantics of counterfactuals in science is none other than the question of scientific representation in disguise. This way the make-believe view of scientific counterfactuals closes the gap between standard philosophical theories of counterfactuals and scientific practice, and reclaims scientific counterfactuals as a rightful object of study for philosophers of science.

#### Extension on previous views

Fictionalist views of counterfactuals have been previously proposed or entertained by, for example, Kim and Maslen (2006), Kimpton-Nye (2020), McLoone (2019) and Wilson (2021). These views have a common kernel: counterfactuals’ antecedents are props in games of make-believe. The make-believe view of scientific counterfactuals shares this kernel, but diverges from the previous views in a number of different respects.

Regarding *motivation*, while **non-vacuity** undoubtedly plays a central role in fictionalist proposals for counterfactuals, fictionalism is here positively considered a genuine alternative to other extant accounts of counterfactuals. In this respect, it differs from the proposals by Kimpton-Nye (2020) or Wilson (2021). They both suggest it as a last resource for the modal necessitarian, for whom counterlegals turn out to be counterpossibles, in order to evade the vacuity thesis the Lewis-Stalnaker semantics push the view into. Furthermore, Kimpton-Nye’s view has somewhat bizarre consequences: the picture painted is one of coexistence between two different semantics for counterlegals depending on one’s metaphysical view on laws—the modal necessitarian may make use of make-believe semantics, while the rest of metaphysicians may resort to possible-worlds semantics as usual. Unfortunately, a semantic dualism of a similar kind also affects the modal necessitarian’s own toolbox, who while encouraged to make use of fiction when faced with counterpossibles, still makes use of possible-worlds semantics when faced with regular counterfactuals.^29^ This is a strange place to draw the line between fiction and non-fiction, especially considering that, as highlighted in Sect. 4.1.2, it might sometimes be complicated to judge whether a counterfactual is a counternomic.^30^

Regarding *scope*, the make-believe view of scientific counterfactuals also differs from the views by Kimpton-Nye (2020) and Wilson (2021) in that it does not apply exclusively to counterpossibles. While I have partly motivated my view, via **non-vacuity**, as a strategy that is able to accommodate both counternomics and countermetaphysicals in science, I have also argued it is in principle applicable to all scientific counterfactuals. Recall that the notion of fiction that make-believe exploits is *not* fiction understood as falsity but rather fiction as imagination, and that evaluation via make-believe is independent of one’s beliefs about the (im)possibility of the state of affairs described by a counterfactual’s antecedent (Stuart et al., 2022; Byrne, 2022). These are observations which challenge the existence of any constitutive relationship between make-believe and nomological or metaphysical impossibilities. With respect to scope, the make-believe of scientific counterfactuals is a narrower version of Kim and Maslen (2006)’s make-believe view of counterfactuals simpliciter. However, their proposal does not dwell on anything like **connection to models**.^31^ McLoone (2019) does make this link to this desideratum, by saying that “a modeler engages in a game of counterfactual make-believe when considering a model” (p. 670). However, he proposes an inverse relation between the two (namely, from models to counterfactuals), and his proposed fictionalism extends only to formal models.^32^

Finally, regarding *morals*, the make-believe view of scientific counterfactuals highlights a new angle from which to evaluate counterfactuals in science: via the prism of scientific representation. I don’t claim it is a better view of counterfactuals in science—scientific representation is yet another thorny subject—but it is definitely one that is better connected to how actual scientific practice works. After all, it is model-world similarities, and not similarity relations across possible worlds, what scientists talk about.

#### Case studies

In what follows, l show how the make-believe view of scientific counterfactuals applies to a couple of counterfactuals from Sect. 2. Let us start by looking at IDEAL PENDULUM:

- **IDEAL PENDULUM:** “If pendulum *X* (an actual pendulum) were a simple pendulum, then for small swings its period *T* would only depend on its length *l* and the gravitational acceleration *g*.” (Godfrey-Smith, 2009, p. 167)

In this case, the prop is the counterfactual’s antecedent. Given that it clearly refers to the ideal model of the simple pendulum, we can consider the description of said model to be (the relevant) part of the prop. Now, what does said description include? In the first place, the pendulum’s equation of motion, the differential equation:

2 $$\begin{aligned} \frac{d^{2}{\theta }}{dt^{2}} + \frac{g}{l}sin\theta = 0 \end{aligned}$$

Note that Eq. 2 in its own does not constitute a model; it only becomes a simple pendulum model when its variables are given an interpretation, i.e. when these are construed as representing properties of a real-world system. In this case, *l* is the length of the string, $\theta$ the angular displacement from the vertical to the pendulum, and *g* is the local acceleration of gravity acting on the bob.^33^

Other things that are part of the model’s description and hence the prop are the assumptions made when constructing said model: the string is massless, the bob is a point mass, there is no air resistance, etc. Finally, one might include a visualisation of said model which facilitates comprehension of the model’s features, such as the following:

**Fig. 1:**
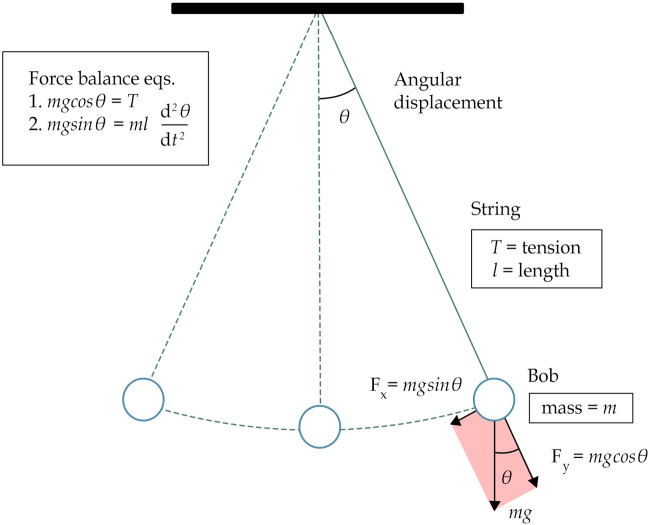
Simple pendulum representation

In short, the relevant part of the prop is the description of the simple pendulum model, which includes: the equation of motion, the information of what property of real-world systems each variable stands in for, the assumptions that went into the model, and potentially a visualisation like Fig. 1.

Next, there is the question of what the principles of generation for this game of make-believe are. One principle of generation clearly at play in this case is the mathematical knowledge of the Taylor series that for small $\theta$s (no more than $15^\circ$), $\sin \theta \approx \theta$—what is called the ‘small angle approximation.’^34^ In this regime Eq. 2 becomes an analytically solvable second-order differential equation analogous to the one for the simple harmonic oscillator. Furthermore, by adding in the small angle approximation, another principle of generation becomes manifest: the physical knowledge that the pendulum’s period, *T*, will then not depend on the angular displacement, as Eq. 3 shows:

3 $$\begin{aligned} T \approx 2\pi \sqrt{\frac{l}{g}} \end{aligned}$$

Equation 3, together with the interpretation of what physical properties the variables stand in for, constitutes another principle of generation at play in this example. Now, when plugging in the simple pendulum model description together with the aforementioned principles of generation, one is prescribed to imagine that ‘... then for small swings its period *T* would only depend on its length *l* and the gravitational acceleration *g*.’ Hence, IDEAL PENDULUM is f-true. Note that here I am assuming the figure of an informed reasoner, who has the epistemic resources—i.e. has access to the right principles of generation—to successfully pretend the imaginings triggered by IDEAL PENDULUM.

Having examined in detail an application of the fiction view of scientific counterfactuals to a counterfactual which clearly invokes a familiar model, I would now like to turn to a less crystal-clear example. Consider the following counterfactual:

- **H-BONDS:** “If water had not had intermolecular hydrogen bonding, then it would have been a gas at room temperature.” (adapted from Tan, 2017, p. 959).

**Fig. 2:**
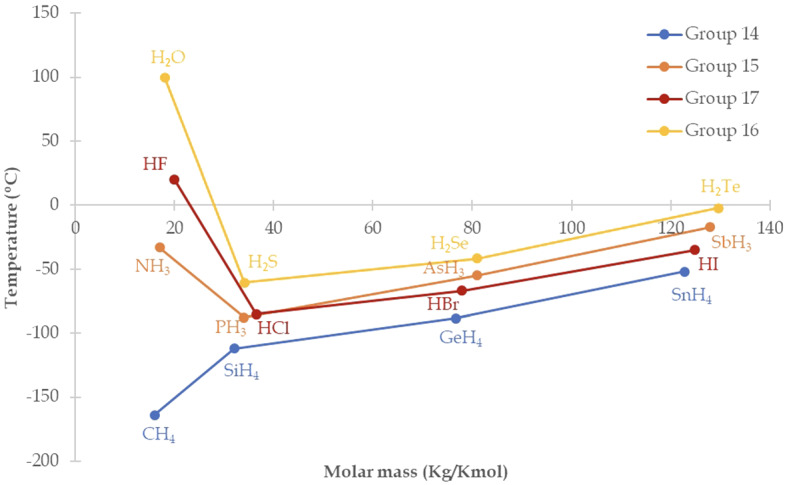
Relation of boiling points ($^\circ \hbox {C}$) versus molar mass (Kg/Kmol) for molecules in hydrides’ groups 14, 15, 16, and 17

One might argue that H-BONDS looks less intuitively connected to a model than IDEAL PENDULUM. This is because it doesn’t appeal to a well-established scientific model, yet that is not to say there isn’t one. The evaluation of impossible counterfactuals will often not only require the analysis of a model but also the construction of one (e.g. by interpolating from the data points in Fig. 2 and providing an interpretation of these); this is not strange, since these possibilities are more remote than those expressed by regular counterfactuals, so it should not surprise us that the models that represent them haven’t yet been explored.

Having said that, let us examine H-BONDS. Here the prop is the description of water not bonded via hydrogen bonds. An informed reasoner knows that water’s normal boiling point is $100^\circ$, so it is liquid at room temperature. However, the other molecules of the same group of hydrides, $\text {H}_{2}\text {S}$, $\text {H}_{2}\text {Se}$ and $\text {H}_{2}\text {Te}$ (group 16), are gaseous at room temperature. This is because they are bonded by van der Waals bonds, which are weaker than the dipole-dipole interactions of the hydrogen bonds. Their boiling points, represented by the blue line in Fig. 2, obey a nearly linear relationship with respect to molecular mass, only broken by $\text {H}_{2}\text {O}$’s behaviour.^35^ Extrapolating from this relationship one would expect $\text {H}_{2}\text {O}$’s boiling point to be about − 70 or $-\,80^\circ \hbox {C}$ (Tan, 2017, p. 959).

The almost-linear relationship between molar mass and boiling point for hydrides, as well as the knowledge that all molecules of group 16 except for $\text {H}_{2}\text {O}$ are bonded via van der Waals bonds, are the principles of generation in action in this game of make-believe. Note that here the periodic table, which Weisberg classifies as an instance of ADR (see Sect. 4.2.1), is part of the principles of generation in the game of make-believe induced by this counterfactual’s antecedent. Additionally, given that H-BONDS’ antecedent doesn’t specify an alternative bonding structure for $\text {H}_{2}\text {O}$, we may add in a reality-oriented principle à la Walton which ensures that its alternative bonding is that of its most similar molecules, namely, those in the same group. Via these implied truths, the model is generated.

Under the fiction of H-BONDS’ antecedent, it follows that ‘... then [water] would have been a gas at room temperature.’ H-BONDS is therefore f-true. Does this counterfactual tell us anything about real water? This f-truth is informative beyond the content of the game of make-believe provided one takes the model output by said counterfactual to represent actual water. Here the answer will depend on one’s account of scientific representation, as well as one’s metaphysical commitments. Note that at this point modal questions are brought back into the picture: would anyone argue that it is essential to water that it is bonded in the way it actually is, because this is what gives it its life-sustaining properties, the modal modelling of the sort involved when reasoning about this counterfactual wouldn’t offer any connections between truth-in-fiction and truth. Unfortunately, this discussion is beyond the scope of this paper. Regarding modality, the important point to emphasise is that questions of this sort can be bracketed when evaluating scientific countefractuals, and this is a feature which make-believe successfully captures.

## Conclusion

In this paper I have vindicated the *make-believe view of scientific counterfactuals*. This is a fiction-based view of counterfactuals which has been defended as an account of counterfactuals and counterfactual reasoning in science which takes scientific practice at face value. On the basis that modelling is the keystone of scientific reasoning, I have established **connection to models** as a desideratum for any view which aims to respect scientific practice. The make-believe view of scientific counterfactuals builds upon indirect fiction views of models in the following way: counterfactuals’ antecedents appeal to more or less well-established scientific models, which are props in games of make-believe which may or may not invite the truth of the respective consequents. In this way, games of counterfactual make-believe in science are none other than rational reconstructions of model-building and model-reasoning processes.

What else can be learnt by reflecting upon actual scientific practice? Undeniably, many of the assumptions that go into scientific models describe nomologically or metaphysically impossible states of affairs. In this respect, recent theorising and experimenting from scientifically-minded philosophers and cognitive scientists has defied the vacuity thesis, a well-known result in the Lewis-Stalnaker similarity analysis of counterfactuals. Hence, I have argued that naturalising the study of counterfactuals also requires paying attention to the desideratum of **non-vacuity**. One way to handle nomological and metaphysical impossibilities in counterfactuals is by treating the states of affairs which counterfactuals describe as imaginary. The central role of the imagination in counterfactual reasoning has been highlighted both by cognitive science researchers and philosophers of science. The make-believe view of scientific counterfactuals respects this intuition, because it characterises fiction as the attitude of imaginative engagement Walton called ‘make-believe.’

The make-believe view of scientific counterfactuals characterises the nature of the imagination as propositional and the resulting semantics for counterfactuals in the following way: a counterfactual is fictionally-true iff its consequent is prescribed to be imagined by its antecedent (a model description, and hence satisfying **connection to models**) and by the pertinent principles of generation, which in science are logical rules of inference, mathematical knowledge, and other field-specific theoretical knowledge. The modal status of the state of affairs described by a counterfactual antecedent is not an ingredient of make-believe, so the vacuity thesis can thus be evaded. One positive feature of the present account is precisely this independence of counterfactual evaluation from any particular metaphysics.

Such modal questions, temporarily bracketed during games of make-believe triggered by counterfactuals’ antecedents and the appropriate principles of generation, are foregrounded when one asks whether—and if so, how—a certain model represents the world. Whether the imaginings triggered by games of counterfactual make-believe are true or false outside the fiction is a question our preferred theory of scientific representation shall address. The close connection of scientific counterfactuals with the question of scientific representation via models is precisely the feature which the present account has aimed to highlight, a feature which has been to date significantly overlooked in the study of the meanings of counterfactuals in science. Via make-believe, I urge philosophers of science to reclaim their position in the present debate.

## Footnotes

1 One caveat: because counterlogicals and countermathematicals don’t feature in scientific explanations and models, they fall outside of the current proposal. Hereafter whenever I refer to counterpossibles I will be referring exclusively to countermetaphysicals.

2 Note that counternomics turn out to be countermetaphysicals for those who consider physical structure or laws of nature metaphysically necessary. This point will be discussed in detail in Sect. 3.1.

3 This study counters Dohrn’s objection that the claims of philosophers like Jenny, Tan, and McLoone are not to be taken seriously “because the reconstruction of scientific practice by philosophers is just another piece of philosophical theory and should be treated accordingly” (Dohrn, 2021, pp. 118–119).

4 Even though the similarity approach has been traditionally attributed to these two philosophers, it was developed contemporaneously by a few people (see Starr, 2021, Sect. 2.3). Note that the Lewis-Stalnaker possible-worlds-based semantic analysis is not the only philosophical analysis of counterfactuals on the table. Another well-known analysis is Edgington’s suppositional theory of conditionals (2003, 2008), a Ramsey-inspired view which builds upon Ernest Adams’ logic of conditionals (Adams, 1965). Since the counterpossibles literature has engaged only with the former—for under the latter the vacuity thesis does not obtain—I’ll here focus on the former as well.

5 This is usually the case since Kripke (1980), with only modal realists like Lewis himself envisioning possible worlds in more ontologically substantive terms.

6 Walton calls these propositions ‘silly questions’ (Walton, 1990, pp. 174–182).

7 Dohrn (2021, p. 719) questions the link between informativeness and non-vacuity. He argues that informativeness is tied to counterfactuals being embedded into the right argumentative context, following Williamson (2007, p. 173) and McLoone (2010, p. 258). However, so far it looks like the view that vacuity should be preserved is minoritarian.

8 While I have here focused on the similarity analysis, the criticisms of the vacuity thesis are applicable to other factualist views of counterfactuals. See McLoone (2021) for a critical engagement with Williamson’s defence of the vacuity thesis.

9 Opinions are divided with respect to whether and how necessitarian accounts of laws can provide a realist semantics for counternomics. Handfield (2004) has defended that the dispositional essentialist can evade the vacuity thesis with the help of 2D semantics: e.g. while it is metaphysically necessary that water is a discrete fluid, it is epistemically or conceptually possible that it is continuous. Kimpton-Nye (2020) agrees with Handfield, but argues that his strategy can unfortunately not help the modal necessitarian. Tan (2019, p. 46) believes that differently-structured water—continuous and incompressible water—could not fill the functional and theoretical roles that actual water fills.

10 Byrne also shows that people tend to imagine alternative scenarios to more recent events rather than earlier events, as well as to controllable rather than uncontrollable events.

11 Following the semantic view of theories models might also be understood as mathematical structures to which an interpretation is then given, but this is not the sense of ‘model’ with which I am concerned here; models *as* imagined systems are already representations in themselves.

12 One of the problems of using conditional probability to assess conditionals is that if an antecedent *A* is impossible it has null probability $Pr(A)=0$, so the conditional probability $Pr(B/A)=P(A\cup B)/Pr(A)$ is undefined. Despite this advantage over probabilistic accounts, Evans and Over (2004, p. 124) consider the fact that mental models accommodate counterfactuals as well as indicative conditionals a bug rather than a feature; they argue that mental models have too little representational content, as they cannot represent whether a possibility is very close or very distant to actuality.

13 Closeness to actuality does however constrain the evaluation of their truth-values. In Byrne (2022) she shows that these evaluations differ depending on whether consequents are same/opposite to reality, or their correspondence to reality is unknown.

14 Otherwise, Byrne (2005) argues, its usefulness and reliability would be somewhat mysterious. Her argument for the rationality of the imagination is more elaborate than that, but unfortunately I don’t have the space to discuss it here. It rests on three separate assumptions: (i) the imagination is rational, (ii) human reasoning depends on the imagination of possibilities, and (iii) counterfactual thoughts rely on thinking about possibilities.

15 Recall that ‘actual’ is indexical; which world is considered as actual is designated by the context in which a counterfactual is uttered.

16 Teller (2009) argues that while they are ontologically on a par, they are epistemically different categories.

17 Walton (1990) argued that the sort of imagination required for make-believe is imagining *de se*, where it is described as a form of imagining where one imagines herself “*doing* or *experiencing* something (or *being* a certain way),” i.e. where one is part of the content of what is imagined (p. 29). While imaginings de se and objectual imaginings are compatible with make-believe, given that the focus of this paper is scientific counterfactual reasoning and representation, it is make-believe’s construal as essentially a propositional attitude that makes the application interesting, for it allows us to speak of truth/falsity within games of make-believe. This is not to say that propositional and imagistic imagination may not come together, but as Salis and Frigg (2020) argue, it is the propositional imagination which characterises scientific models and, by extension, as I’ll argue, counterfactuals. Murphy (2020) has attacked this monist characterisation of the scientific imagination by mainly pointing out that thought experiments appeal to different varieties of the imagination. Her criticism thus does not at face value affect the claim that the variety of the imagination which characterises models and counterfactuals is propositional.

18 See Sect. 4.2 for an explanation of how fictional truth might map onto truth.

19 Note that Godfrey-Smith holds an indirect fiction view of models but rejects make-believe; he criticises Frigg’s account on the basis that comparisons between the uninstantiated properties of model systems and the real properties of target systems are problematic (Godfrey-Smith, 2009, p. 113; 2020, p. 161)

20 Another viable inclusive alternative is to reconceptualise the indirect view of models into the ‘new fiction view of models’ (Salis, 2020), a view which in order to account for the relationship between the properties attributed to imaginary objects by the model and real objects abandons the notion of a model system and appeals in favour of the notion of a ‘mental file’. Mental files are cognitive structures used by anti-realists about intentionality for the storage of information about an object, which may or may not exist.

21 As Levy (2015) argues, while positing and analysing a model, on the one hand, and assessing its relation to the actual world, on the other, might not come apart in actual practice, they are logically distinct operations. The indirect fiction view recognises the logical distinctness of these two operations.

22 I would like to thank an anonymous reviewer for raising this concern.

23 The operation of these principles is supported by evidence from mental models experiments (Byrne, 2005), which as seen in Sect. 3.2 shows that when devising counterfactual scenarios people tend to imagine worlds with the same natural laws.

24 Thanks to an anonymous reviewer for the suggestion to incorporate the following clarification.

25 *Selectivity* describes the fact that when reasoning counterfactually one selects a particular manner in which things could have been different. To some extent, one could argue that the mutual belief principle targets the explanatory relevance that selectivity aims to track. *Reality orientation* is actually none other than the reality principle: from all the possible models a counterfactual’s antecedent is compatible with, we select the one that is the closest to reality.

26 Thanks to a reviewer for raising this concern, and pointing at the view I subsequently discuss.

27 Furthermore, I worry that the focus on modelers’ intentions doesn’t sit well with targetless systems. Consider the case of aether models: presumably an example of ADR that fell out of steam once special relativity was developed? A focus on the products of theorising seems to better stand the test of time.

28 For an overview of the debate about the epistemic uses of the imagination, see Kind and Kung (2016) and Badura and Kind (2021). For more specific discussion of the epistemic contribution of the scientific imagination, see Levy and Godfrey-Smith (2020). Myers (2021), Friend (2020), and Godfrey-Smith (2020), for instance, agree that the imagination cannot be used as an ultimate source of justification for knowledge.

29 The reason for said encouragement is that “[i]f we were to embrace thoroughgoing fictionalism in the interest of avoiding semantic dualism, [modal necessitarianism] would lose its substance because it would be unclear in virtue of what counterlegal suppositions are different from counterfactual suppositions consistent with the laws, in which case, the claim that the laws are strongly necessary loses its bite” (Kimpton-Nye, 2020, p. 531). While I find this argument compelling, this semantic dualism might as well be read as an invitation to abandon modal necessitarianism.

30 What I have in mind here are counterfactuals for which we have a high credence that they have an almost zero chance of being physically possible, such as those about supersymmetric particles (neutralinos, squarks, sleptons, etc.). Thanks to Mike T. Hicks for raising this point.

31 Arguably, given my minimal characterisation of models, the two views could be made to coexist. However, there is definitely more structure in scientific counterfactual reasoning than in day-to-day counterfactual reasoning, so it would have to be worked out where the differences lie, whether in the nature or in the constraints on make-believe.

32 McLoone clearly thinks that the consequents of mathematical counterfactuals are entailed by their antecedents. However, if what is doing the work is a deductive inference is deduction from the antecedent, it is unclear what role make-believe plays. Furthermore, there is nothing like purely mathematical counterfactuals in science. Consider the example of the well-known Lotka-Volterra predator-prey model from ecology: “If a pair of populations had features *F*, then it would have/do *G*” Godfrey-Smith (2020, p. 168). This is not an instance of mathematical necessity, for information about initial conditions regarding the type and number of prey, predator, as well as the parameters of growth and death rates need to be specified in order to evaluate whether the consequent follows. Even those who view models as essentially abstract mathematical objects, like Weisberg (2013), add an interpretative element to the definition of models.

33 See Salis (2020) for a detailed exposition of this example.

34 Lima and Arun (2006) report an even lower value for the angular displacement. If an error less than 0.1% is desired, the displacement cannot be more than $7^\circ$.

35 Note that the same almost-linear pattern is observed for the other three groups.
